# Bias in vital signs? Machine learning models can learn patients’ race or ethnicity from the values of vital signs alone

**DOI:** 10.1136/bmjhci-2024-101098

**Published:** 2025-07-10

**Authors:** Bojana Velichkovska, Hristijan Gjoreski, Daniel Denkovski, Marija Kalendar, Irene Dankwa Mullan, Judy Wawira Gichoya, Nicole Martinez, Leo Celi, Venet Osmani

**Affiliations:** 1 Ss Cyril and Methodius University in Skopje, Skopje, North Macedonia; 2 Milken Institute School of Public Health, George Washington University, Washington, District of Columbia, USA; 3 Emory School of Medicine, Emory University, Atlanta, Georgia, USA; 4 Stanford University School of Medicine, Stanford University, Stanford, California, USA; 5 Institute of Medical Engineering and Science, Massachusetts Institute of Technology, Boston, Massachusetts, USA; 6 Division of Pulmonary, Critical Care and Sleep Medicine, Beth Israel Deaconess Medical Center, Boston, MA, USA; 7 Harvard T.H. Chan School of Public Health, Harvard University, Boston, MA, USA; 8 Digital Environment Research Institute, Queen Mary University of London, London, UK

**Keywords:** Artificial intelligence, Electronic Health Records, Health Equity, Decision Support Systems, Clinical

## Abstract

**Objectives:**

To investigate whether machine learning (ML) algorithms can learn racial or ethnic information from the vital signs alone.

**Methods:**

A retrospective cohort study of critically ill patients between 2014 and 2015 from the multicentre eICU-CRD critical care database involving 335 intensive care units in 208 US hospitals, containing 200 859 admissions. We extracted 10 763 critical care admissions of patients aged 18 and over, alive during the first 24 hours after admission, with recorded race or ethnicity as well as at least two measurements of heart rate, oxygen saturation, respiratory rate and blood pressure. Pairs of subgroups were matched based on age, gender, admission diagnosis and disease severity. XGBoost, Random Forest and Logistic Regression algorithms were used to predict recorded race or ethnicity based on the values of vital signs.

**Results:**

Models derived from only four vital signs can predict patients’ recorded race or ethnicity with an area under the curve (AUC) of 0.74 (±0.030) between White and Black patients, AUC of 0.74 (±0.030) between Hispanic and Black patients and AUC of 0.67 (±0.072) between Hispanic and White patients, even when controlling for known factors. There were very small, but statistically significant differences between heart rate, oxygen saturation and blood pressure, but not respiration rate and invasively measured oxygen saturation.

**Discussion:**

ML algorithms can extract racial or ethnicity information from vital signs alone across diverse patient populations, even when controlling for known biases such as pulse oximetry variations and comorbidities. The model correctly classified the race or ethnicity in two out of three patients, indicating that this outcome is not random.

**Conclusion:**

Vital signs embed racial information that can be learnt by ML algorithms, posing a significant risk to equitable clinical decision-making. Mitigating measures might be challenging, considering the fundamental role of vital signs in clinical decision-making.

**WHAT IS ALREADY KNOWN ON THIS TOPIC**

- Bias in treatment plans, healthcare providers’ attitudes and clinical scores across race or ethnicity is well documented. However, far less is known about racial or ethnic bias in routinely collected vital signs which are essential in clinical decision-making.

**WHAT THIS STUDY ADDS**

- Our study found that algorithms can learn self-identified race or ethnicity of patients from the values of four vital signs alone, even when controlling for known factors such as pulse oximetry variations and comorbidities.

**HOW THIS STUDY MIGHT AFFECT RESEARCH, PRACTICE OR POLICY**

- We highlight the critical need to raise awareness of unexpected sources of bias in clinical decisions and to rigorously evaluate ML models to prevent perpetuation or amplification of health inequalities.

## Introduction

 Machine learning (ML) algorithms are evolving to tackle increasingly complex clinical challenges.^1^ The general appeal is that clinical practice will likely benefit from algorithm-assisted decision-making by optimising clinical workflows, diagnostic interventions and enhancing personalised precision care. Some insights derived from algorithms will likely assist in clinical decision-making where patients’ lives are at risk. Therefore, ML algorithms that become integrated as part of decision-making in clinical practice must be robust, reliable and unbiased.

Healthcare disparities resulting from discrimination and bias are pervasive. These can be encoded in algorithms trained on clinical data from electronic health records (EHRs). Existing inequities can be perpetuated or even magnified by algorithms developed to inform decision-making due to bias in data used for training the models, bias introduced during model development or deployment and postdeployment monitoring. Any of these can result in decision-making that can be discriminatory and harmful to socially disadvantaged population groups inadequately represented in the data.

There is overwhelming evidence that race or ethnicity impacts clinical decision-making.^2^ Hispanic patients seen by non-Hispanic providers received breast and colorectal cancer screening at higher rates than Hispanic patients seen by Hispanic providers.^3^ Greenwood and colleagues reported a 58% reduction in mortality of Black newborns when under the care of Black physicians compared with White physicians.^4^ Despite reporting greater pain and pain-related disability, minority patients are more likely to receive inadequate pain treatment compared with White patients.^5 6^ Treatment variation across race or ethnicity not explainable by patient or disease factors has been detailed in several studies, accompanied by evidence of unconscious bias in healthcare providers’ attitudes, expectations and behaviour.^7^,^9^ The presence of this type of bias in medical practice is further amplified if the discriminatory attitudes and behaviours are in turn modelled as disease mechanisms or decision support algorithms implemented by care providers. Brooks describes this phenomenon in an opinion piece that frames unconscious bias as a ‘silent curriculum’.^10^ Furthermore, a recent study illustrates racial bias in patients’ EHRs, showing that Black patients are 2.5 times more likely to have one or more negative descriptors compared with White patients.^11^

Bias embedded in data has been illustrated by Obermeyer *et al* where ‘at a given risk score, Black patients are considerably sicker than White patients, as evidenced by signs of uncontrolled illnesses’. The algorithm learnt to predict care costs, placing Black patients in the same risk category as a subset of White patients, while having considerably worse symptoms.^12^ To add to the severity of the problem, the number of Black patients who should have been referred for complex care was halved. Racial disparities have also been observed in blood pressure rates, with Black patients having higher blood pressure.^13 14^ Moreover, patients with darker skin colour are at greatest risk of hypovitaminosis D, which may result in microvascular endothelial dysfunction.^15^

With evidence of racial and ethnic bias in the intuition and judgement of healthcare providers, concern exists that algorithms trained to predict and optimise outcomes may use self-identified racial or ethnic information to inform decision-making even when these parameters are not used during training.

While bias in clinical scores is well-documented, less is known about bias in routinely collected, essential information for clinical decision-making, namely, vital signs. We investigate whether self-identified race or ethnicity can be learnt from four vital signs alone. We will use the term *ethnicity* to refer to self-identified race or ethnicity (https://www.ethnicity-facts-figures.service.gov.uk/style-guide/writing-about-ethnicity/).

Our results show models can predict patients’ ethnicity with an area under the curve (AUC) of 0.74 (±0.030) between White and Black patients, AUC of 0.74 (±0.030) between Hispanic and Black patients and AUC of 0.67 (±0.072) between Hispanic and White patients. If sensitive attributes are easily learnt from essential clinical data, it is of significant concern whether they can become an embedded part of clinical decision-making and treatment optimisation, leading to patient harm.

Our findings add to the growing body of evidence pointing to structural bias in healthcare systems, where even seemingly objective physiological data can perpetuate inequities. Another important interpretation of this finding is that physiological data might present a biased substrate for ML models. This dual interpretation emphasises the need for caution in using ML for clinical decision-making and the potential for physiology data to embed racial and ethnic biases, often rooted in how measurement devices are designed, tested or used in clinical settings. These biases can carry over into ML models trained on this data, further amplifying disparities in healthcare outcomes if not carefully addressed.

## Methods

### Clinical data sources and study population

We used the eICU Collaborative Research Database (eICU-CRD),^16^ containing 200 859 admissions collected from 335 ICUs across 208 hospitals in the USA, admitted between 2014 and 2015. The study uses four vital signs: heart rate, respiratory rate, non-invasive and invasive blood pressure (systolic, diastolic and mean) and oxygen saturation. The cohort selection process is detailed in online supplemental appendix 1. Including the three dominant ethnic groups resulted in a total of 10 763 patient stays, of which 9215 (85%) were White, 1066 (10%) were Black and 482 (5%) were Hispanic. Significantly less data were available for Asian American and Native American patients (177 and 84, respectively), thus they were excluded from this study.

### Matched cohorts

As our patient cohort consisted predominantly of White patients, we devised several matched cohorts based on admission diagnosis, gender, age and Acute Physiology and Chronic Health Evaluation (APACHE IV) score to equally represent the three population subgroups considered in our study (Black, Hispanic and White patients). The process is detailed in online supplemental appendix 2. The rigorous matching and the fact that only 10% of the patients in the original dataset are Black reduced the effective sample size markedly. However, relaxing matching requirements would have made the research less robust.

### Statistical analysis

Patient characteristics were analysed using medians (IQRs) for continuous and frequencies (percentages) for categorical variables. We used the Kruskal-Wallis test (one-way ANOVA) for continuous variables and the χ^2^ test for categorical variables to compare different ethnic subgroups. Due to the selection criteria (online supplemental appendix 1), no patients with missing data remained. Correlations between variables are shown in online supplemental appendix 3.

### Model development and validation

We analysed binary outcomes, namely whether vital signs can predict ethnicity of Black versus White, Hispanic versus White and Hispanic versus Black patients. We used three ML algorithms to derive the models and evaluate performance: Logistic Regression (LR), Random Forest (RF) and XGBoost.^17^ As XGBoost is prone to overfitting, we evaluated two versions of XGBoost: with default and optimised parameters selected using random search.^18^ Details on optimisation are presented in online supplemental appendix 4. We also considered a shallow neural network, with no performance improvement.

We used stratified fivefold cross-validation to evaluate each model, meaning data were divided into fivefolds; each fold maintained the original distribution class-wise. Model derivation was performed on fourfolds, while the remaining fold was used for validation. We repeated the process five times and averaged the final results. We assessed the performance of our models by computing area under the receiver operating characteristic curve (AUC), while we are also aware of analyses to measure performance in multiple subgroups.^19^ This study followed the Strengthening the Reporting of Observational Studies in Epidemiology (STROBE) reporting guidelines.^20^

## Results

Out of 10 763 patients, 1688 met the inclusion criteria for the first matched cohort (Black and White), 784 for the second cohort (Hispanic and White) and 444 for the third cohort (Hispanic and Black). Table 1 shows patient baseline characteristics for each matched cohort. Initially, we investigated prediction of ethnicity using heart rate, oxygen saturation, respiratory rate and blood pressure (systolic, diastolic and mean arterial pressure measured through arm cuff as well as invasively). Then, we performed sensitivity analysis, focusing on patients with comorbidities.

**Table 1:** Patient characteristics for each of the matched cohorts

Clinical values	Black and White matched patient cohort		Hispanic and White matched patient cohort		Hispanic and Black matched patient cohort	
	Black	White	Hispanic	White	Hispanic	Black
Patients	844	844	392	392	222	222
Gender (male)	457 (51.7%)	483 (57.23%)	236 (60.2%)	238 (60.7%)	141 (63.5%)	127 (57.21%)
Age	59 (51, 69) (19, 89)	63 (56, 73)(18, 89)	61 (53, 73)(18, 89)	64 (57, 73)(23, 89)	62 (54, 73)(18, 89)	60 (52, 70)(22, 88)
Heart rate	87 (75, 99)(0, 256)	84 (73, 97)(0, 242)	84 (73, 97)(0, 205)	83 (73, 96)(0, 217)	84 (74, 96)(0, 189)	86 (76, 98) (0, 300)
Invasive oxygen saturation	97.6 (95, 99)(17, 100)	97.4 (95, 99)(12, 100)	97 (95, 99)(43, 100)	97 (95, 99)(28, 100)	97 (95, 99)(54, 100)	97 (95, 99)(27, 100)
Oxygen saturation (pulse oximetry)	99 (97, 100)(0, 100)	98 (95, 99)(2, 100)	98 (96, 100)(15, 100)	97 (95, 99)(15, 100)	98 (96, 100)(35, 100)	99 (96, 100)(0, 100)
Respiration rate	19 (15, 24)(0, 197)	19 (16, 23)(16, 189)	19 (16, 23)(0, 140)	19 (15, 23)(0, 147)	19 (16, 23)(0, 140)	19 (15, 24)(0, 152)
Invasive systolic BP	123 (107, 141)(0, 300)	120 (106, 138)(0, 300)	122 (108, 138)(0, 300)	123 (108, 140)(0, 300)	119 (106, 135)(0, 300)	122 (108, 140)(0, 300)
Invasive diastolic BP	61 (53, 70)(0, 300)	58 (50, 66)(0, 300)	59 (51, 68)(0, 300)	59 (51, 68)(0, 300)	58 (51, 67)(0, 300)	60 (53, 68)(0, 300)
Invasive mean BP	80 (72, 91)(0, 300)	78 (69, 87)(0, 300)	79 (70, 90)(0, 300)	79 (70, 90)(0, 300)	78 (69, 88)(0, 300)	80 (72, 89)(0, 300)
Systolic BP	121 (105, 139)(20, 287)	118 (103, 135)(21, 287)	120 (106, 135)(23, 270)	119 (105, 136)(28, 257)	117 (104, 133) (23, 270)	123 (106, 141)(24, 286)
Diastolic BP	66 (57, 76)(3, 224)	63 (55, 73)(0, 234)	64 (55, 74)(10, 233)	63 (55, 73)(0, 219)	62 (54, 72)(10, 181)	65 (56, 75)(10, 210)
MAP	82 (72, 94)(9, 229)	79 (69, 91)(14, 269)	78 (69, 89)(16, 238)	80 (70, 91)(18, 224)	76 (68, 87)(18, 188)	82 (72, 94)(16, 220)

We show the gender distribution for each self-identified race or ethnicity, accompanied by the percentage of male patients. The remaining characteristics are described by the mean and the first and the third quartiles in the brackets. The second row shows the minimum and the maximum values of the variables. The p values for the variables (where applicable) are shown in online supplemental appendix 5.

BP, blood pressure; MAP, mean arterial pressure.

### Vital signs as a source of bias

**Figure 1:**
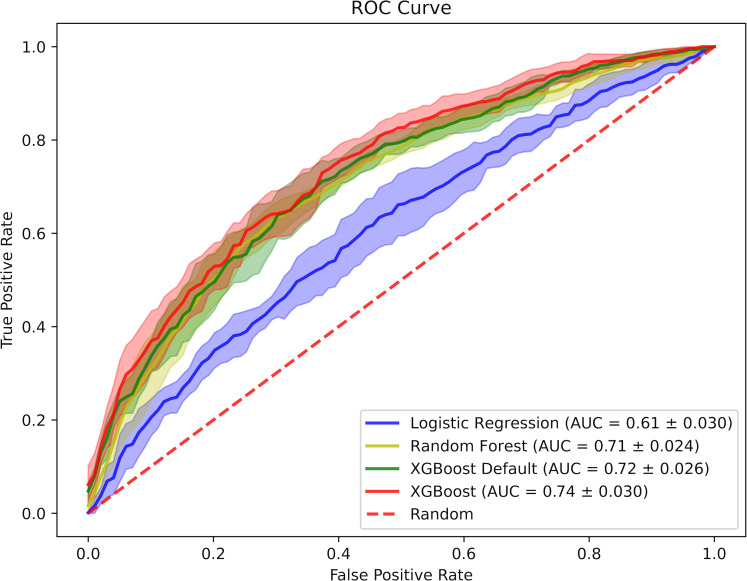
Performance of ML models in predicting patients’ self-identified race or ethnicity using vital signs only as input for Black and White patients. Analysis is based on Logistic Regression, Random Forest, XGBoost with default parameters and XGBoost with optimised parameters found using random search. AUC, area under the curve; ML, machine learning; ROC curve, receiver operating characteristic curve.

We investigated the presence of bias in the overall patient cohort and used this as baseline. Our analysis reveals XGBoost can predict Black and White patients’ ethnicity using only vital signs with a performance of AUC of 0.74 (±0.030) as seen in figure 1 and table 2. Previous work has shown that confounding variables, such as gender and age, can significantly impact the prediction of race or ethnicity.^21^ Therefore, to investigate the effect of potentially confounding variables, we derived a cohort of 1064 patients matched on age using exact matching, rather than the ±5 years range originally used. This resulted in a comparable AUC of 0.78 (±0.019), indicating that even in cohorts with no statistical difference in age and gender, the algorithms can still distinguish patients’ ethnicity.

**Table 2:** Self-identified race or ethnicity prediction performance for all matched cohorts using Logistic Regression, Random Forest, XGBoost and XGBoost with optimised (opt) hyperparameters

	Black and White patients(n=844 per group)	Hispanic and White patients(n=392 per group)	Hispanic and Black patients(n=222 per group)
Logistic Regression	0.61±0.030	0.61±0.048	0.70±0.057
Random Forest	0.71±0.024	0.69±0.069	0.75±0.084
XGBoost	0.72±0.026	0.68±0.080	0.78±0.084
XGBoost (opt)	0.74±0.030	0.67±0.072	0.74±0.030

Results are shown using area under the receiver operating characteristic curve along with SD.

Furthermore, similar performance in predicting patients’ ethnicity also holds when considering patients with comorbidities, as shown in table 3. Only patients with heart failure had significant result deviation.

**Table 3:** Self-identified race or ethnicity prediction performance for the Black and White cohort in patients with comorbidities using Logistic Regression, Random Forest, XGBoost and XGBoost with optimised (opt) hyperparameters

	Sepsis	Essential hypertension	Heart failure	Acute kidney failure	Chronic kidney disease
Logistic Regression	0.57±0.176	0.62±0.073	0.53±0.113	0.60±0.049	0.57±0.099
Random Forest	0.69±0.099	0.62±0.083	0.57±0.059	0.69±0.075	0.69±0.095
XGBoost	0.69±0.093	0.60±0.064	0.51±0.050	0.71±0.068	0.72±0.079
XGBoost (opt)	0.71±0.077	0.60±0.091	0.50±0.063	0.69±0.081	0.67±0.064

Results are shown using area under the receiver operating characteristic curve along with SD.

**Figure 2:**
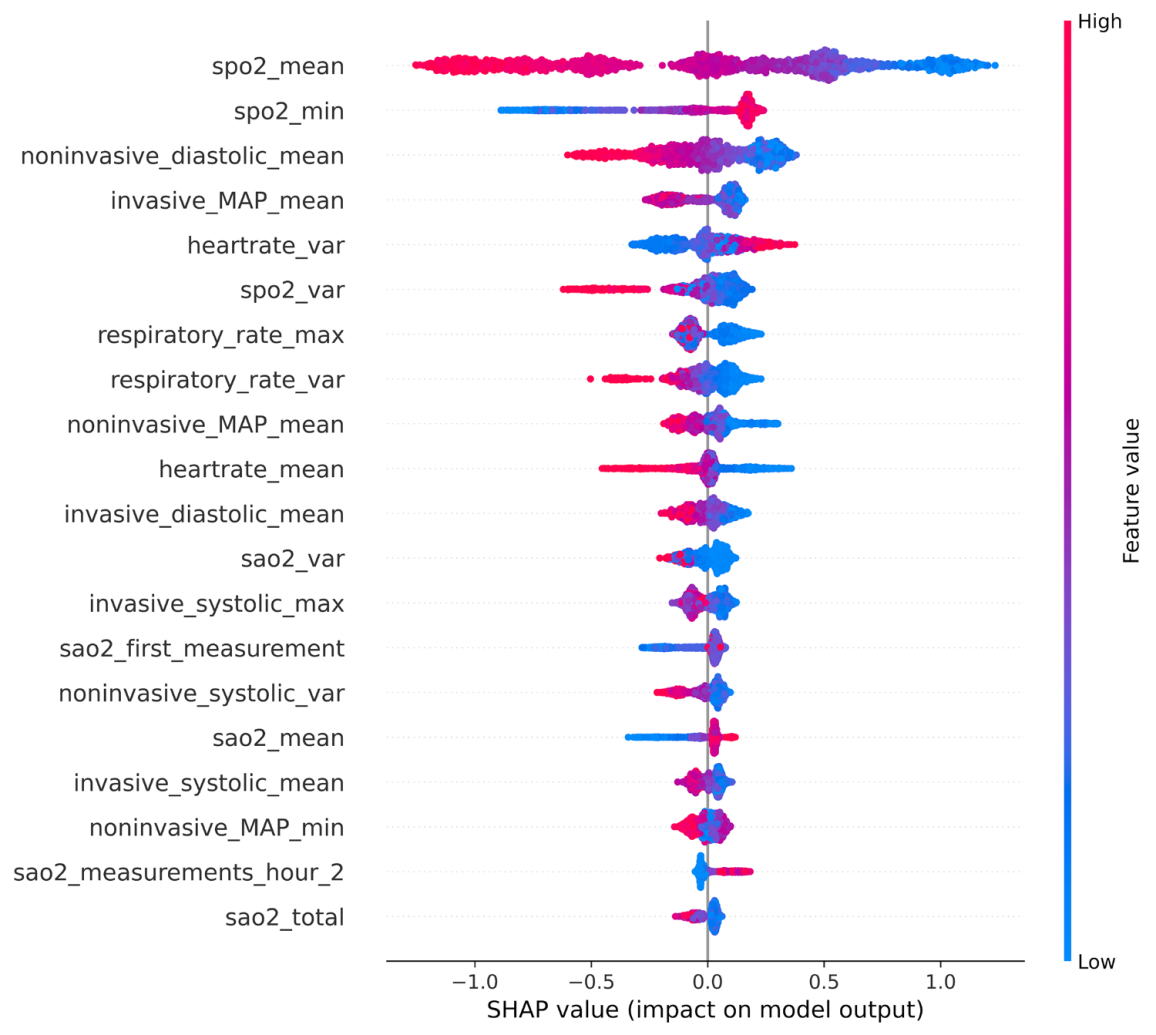
Importance of variables in predicting patients’ self-identified race or ethnicity using all the four vital signs in Black and White patients. Variables are shown in order of importance from the top, with pulse oximetry being the most influential in predicting patients’ race or ethnicity. The variable name suffix indicates whether mean (_mean), minimum (_min), maximum (_max) or variance (_var) value was used for a particular vital sign. The frequencies of measurement were considered in the entire 24 hours (_total), during each of the first 6 hours, after admission (hour_[1–6]). The maximum measurements taken during a period of 1 hour are labelled as maximum_in_24_hours. The red colour indicates higher values of a variable, whereas blue indicates a lower value. SHAP, Shapley Additive exPlanations.

Considering these results, we then probed possible origins of racial information in vital signs through variable saliency analysis. We used the Shapley Additive exPlanations (SHAP) method to understand the influence of each vital sign in predicting patients’ ethnicity. The SHAP analysis (figure 2) revealed that oxygen saturation measured through pulse oximetry was the most influential variable in predicting patients’ ethnicity. Therefore, we focused on investigating not only pulse oximetry, but also technological approaches used to measure vital signs in general as potential sources of racial information.

### Technologies used to measure vital signs as a potential source of racial information

We investigated whether devices used to measure vital signs can be a source of ethnic bias since earlier work has shown that skin colour differences can influence pulse oximetry readings.^22^ Furthermore, acquiring accurate readings of blood pressure using an arm cuff is challenging in patients with high BMI.^23^ To see whether ML algorithms could pick up these known differences, we divided the analysis into investigating potential racial information in (1) blood pressure measurements using an arm cuff and (2) oxygen saturation measurements using pulse oximetry.

#### Racial information in blood pressure values measured using an arm cuff

We compared the performance of ethnicity prediction using blood pressure values measured through an arm cuff (non-invasive) with that of an arterial line (invasive), in addition to using both types of measurements. For these tests, we consider heart and respiration rates, as well as invasive and non-invasive blood pressure measurements. Oxygen saturation features were disregarded.

In contrast to existing literature, our results showed that while there is a presence of racial information in blood pressure measurements irrespective of the measurement method used (AUC of 0.63±0.035), we did not find major differences between values measured using an arm cuff (AUC of 0.64±0.036) in comparison to the values measured through an arterial line (AUC of 0.63±0.025). Results are summarised in table 4.

**Table 4:** Self-identified race or ethnicity prediction performance results for different potential sources of bias using Logistic Regression, Random Forest, XGBoost and XGBoost with optimised (opt) hyperparameters

	Non-invasive blood pressure	Invasive blood pressure	Non-invasive and invasive blood pressure	Non-invasive oxygen saturation	Invasive oxygen saturation	Non-invasive and invasive oxygen saturation	Frequency of measurements of vital signs	Other sources of bias
Logistic Regression	0.61±0.032	0.58±0.027	0.59±0.024	0.73±0.033	0.59±0.032	0.72±0.032	0.55±0.043	0.62±0.044
Random Forest	0.62±0.030	0.62±0.038	0.61±0.025	0.70±0.030	0.59±0.021	0.69±0.025	0.56±0.028	0.62±0.035
XGBoost	0.59±0.030	0.57±0.016	0.59±0.027	0.67±0.027	0.58±0.021	0.68±0.027	0.55±0.030	0.59±0.029
XGBoost (opt)	0.64±0.036	0.63±0.025	0.63±0.035	0.72±0.028	0.60±0.023	0.72±0.030	0.57±0.019	0.64±0.034

Results are shown using area under the receiver operating characteristic curve along with SD.

#### Racial information in oxygen saturation values measured using pulse oximetry

We also investigated the presence of racial information in oxygen saturation stemming from measurement technologies by comparing the performance of ethnicity prediction using oxygen saturation values obtained from pulse oximetry (non-invasive) with those obtained from an arterial line (invasive). For these tests, we considered heart and respiration rates and invasive and non-invasive oxygen saturation measurements. Blood pressure features were disregarded.

Our findings showed that pulse oximetry is a source of racial information with an AUC of 0.72 (±0.028). This is in severe contrast with results obtained from invasively measured oxygen saturation (arterial line), where these values predict patients’ ethnicity with an AUC of 0.60 (±0.023), as shown in table 4. Our results support previous findings that pulse oximetry is affected by skin colour.^22^

### Racial information in care delivery practices reflected in vital signs

Additionally, we focused on care delivery practices as reflected in the frequency of measurements of vital signs, rather than actual values. We find care delivery practices do not significantly influence the prediction of patients’ ethnicity with an AUC of 0.57 (±0.019), as shown in table 4. This may also be because vital signs are measured far more routinely than other variables, and consequently, if bias indeed exists, it would be difficult for the algorithms to ascertain.

### Other sources of racial information reflected in patients’ vital signs

Finally, we investigated whether ethnicity information can be learnt when controlling for measurement technologies and care delivery practices. For this analysis, we used values of invasively measured vital signs only. This is because invasively measured vital signs are less prone to being influenced by measurement technology. Even when controlling for these factors, we show that ethnicity can be learnt from vital signs with an AUC of 0.64 (±0.034), detailed in table 4.

It is difficult to pinpoint potential sources of ethnicity. One hypothesis could be calibration differences in APACHE IV scores as shown in Sarkar *et al*,^24^ or differences in disease severity not being reflected in APACHE IV scores.^12^ However, upstream factors, such as patient selection criteria for an arterial line or even availability of patients might have also contributed to ethnicity being embedded in vital signs.^25^

## Discussion

We have shown that ML algorithms can learn self-identified racial information from vital signs alone. This is unexpected as racial information was not thought to be present within vital sign values. Furthermore, the ability of ML algorithms to learn ethnicity from vital signs generalised to diverse patient populations and held even when controlling for known sources of racial or ethnic bias, such as pulse oximetry readings affected by skin colour, care delivery practices, which were not found to contribute to racial information, and presence of comorbidities.

While a definite prediction of ethnicity cannot be obtained, the model’s success in correctly classifying two out of three patients is not accidental. Our models use information from patients’ vital signs only, suggesting that statistical features from routinely collected information in the first 24 hours of admission contain embedded information along racial dimensions. Pulse oximeter readings, considered an important unbiased measure of hypoxaemia, were shown to be influenced by skin colour, which came to light during the COVID-19 crisis.^22 26 27^ On further investigation, it was revealed that oxygen saturation levels had greater variability in patients who identified as Black, followed by Hispanic, Asian American and least in White patients. This showcases an important source of bias stemming from neglect, that is, the lack of rigour in technology development, even in sensitive areas such as healthcare. While our saliency analysis showed pulse oximetry as an important variable, its correlation with ethnicity does not fully explain our findings. These results show this bias propagates even after data aggregation, thus further emphasising the need for technological improvement.

In addition to the potential risks, our study also highlights challenges in mitigation measures. A common approach, although not without critics,^28^ is to selectively remove variables that encode sensitive attributes, so ML models do not learn from them and consequently sensitive information does not become part of the decision process. Ubiquitous use of vital signs in clinical decision-making renders this approach impossible, not least because the origin of racial information appears to be difficult to isolate. Perhaps the time has come to apply a counter approach, by using sensitive attributes such as race or ethnicity to facilitate audit for possible algorithmic bias and adapt established policies on how to ethically collect, use and report data on race or ethnicity.^29^ Moreover, one of the biggest challenges of the study stemmed from limited or missing information in the dataset. Therefore, our selection process illustrates the need for larger and more encompassing datasets, which contain relevant variables such as race or ethnicity, gender and age detailed with diversity necessary to make them all-inclusive.

### Limitations

While our study includes a well-studied, large and diverse patient population, allowing investigation of bias for several racial groups, some limitations exist. Use of self-identified race presents a challenge, as studies have shown that genetic variability is higher within the races than between the races,^30^ rendering race more a social construct rather than a biological one. Following on, race or ethnicity in this study included rigid categories that did not account for patients of mixed ancestry as well as limited availability of data from other racial identity categories. We included Black, Hispanic and White patients only. Other racial, ethnic identities (namely Asian American and Native American patients) had insufficient data for a robust analysis. Furthermore, because of the rigorous cohort matching and that only 10% of the patients in the original dataset are Black, the effective sample size is markedly reduced. However, relaxing the requirements for matching would have made our investigation less robust, while observational studies, such as ours, allow us to further refine the hypothesis for follow-up studies. Finally, our study focused on the US-based patient population; therefore, further investigation would be required to determine whether these results are generalisable to centres outside of US-based ICUs.

These limitations highlight the need for more inclusive and representative datasets in future research. This could be achieved through targeted data collection in collaboration with national registries or institutions working with under-represented populations as a means of ensuring broader demographic coverage—including individuals of mixed ancestry and racial groups beyond Black, Hispanic and White populations.

## Conclusion

As ML weaves itself into the fabric of healthcare, there is increasing attention on the effect of algorithms on under-represented, marginalised or disadvantaged populations. Algorithms used to identify patients with complex health needs were found to perpetuate racial disparities, leading to a call for greater algorithmic transparency by the US Senate. Our work, while in the same vein, goes beyond this call by additionally drawing attention to unexpected sources of bias and the potential harm, given their ubiquitous use in clinical decision-making.

## Supplementary material

10.1136/bmjhci-2024-101098 online supplemental file 1

## Data Availability

Data are available in a public, open access repository.
