# Supplementary material for: Bias in vital signs? Machine learning models can learn patients’ race or ethnicity from the values of vital signs alone
Source: BMJ Health Care Inform. 2025 Jul 10;32(1):e101098. doi: 10.1136/bmjhci-2024-101098 (PMC12258377; doi:10.1136/bmjhci-2024-101098)
Supplement: online supplemental file 1 [file bmjhci-32-1-s001.docx]

# Appendix 1 – Cohort selection diagram

We selected all adult patients (age 18 and over) alive within the first 24 hours after admission with at least two clinically valid measurements for all vital signs considered for the study: heart rate, respiratory rate, non-invasive and invasive blood pressure (systolic, diastolic, and mean) and oxygen saturation. There were insufficient measurements of temperature. We selected data from the first 24 hours, given that patients’ presenting vital signs inform follow-up and life-saving interventions. Consequently, potentially biased data during this critical period can cause the most harm. We extracted mean, minimum, maximum, and variance for each vital sign. For the requirements of the matching process, patients with missing APACHE IV, admission diagnosis, age, or gender were excluded from the study.


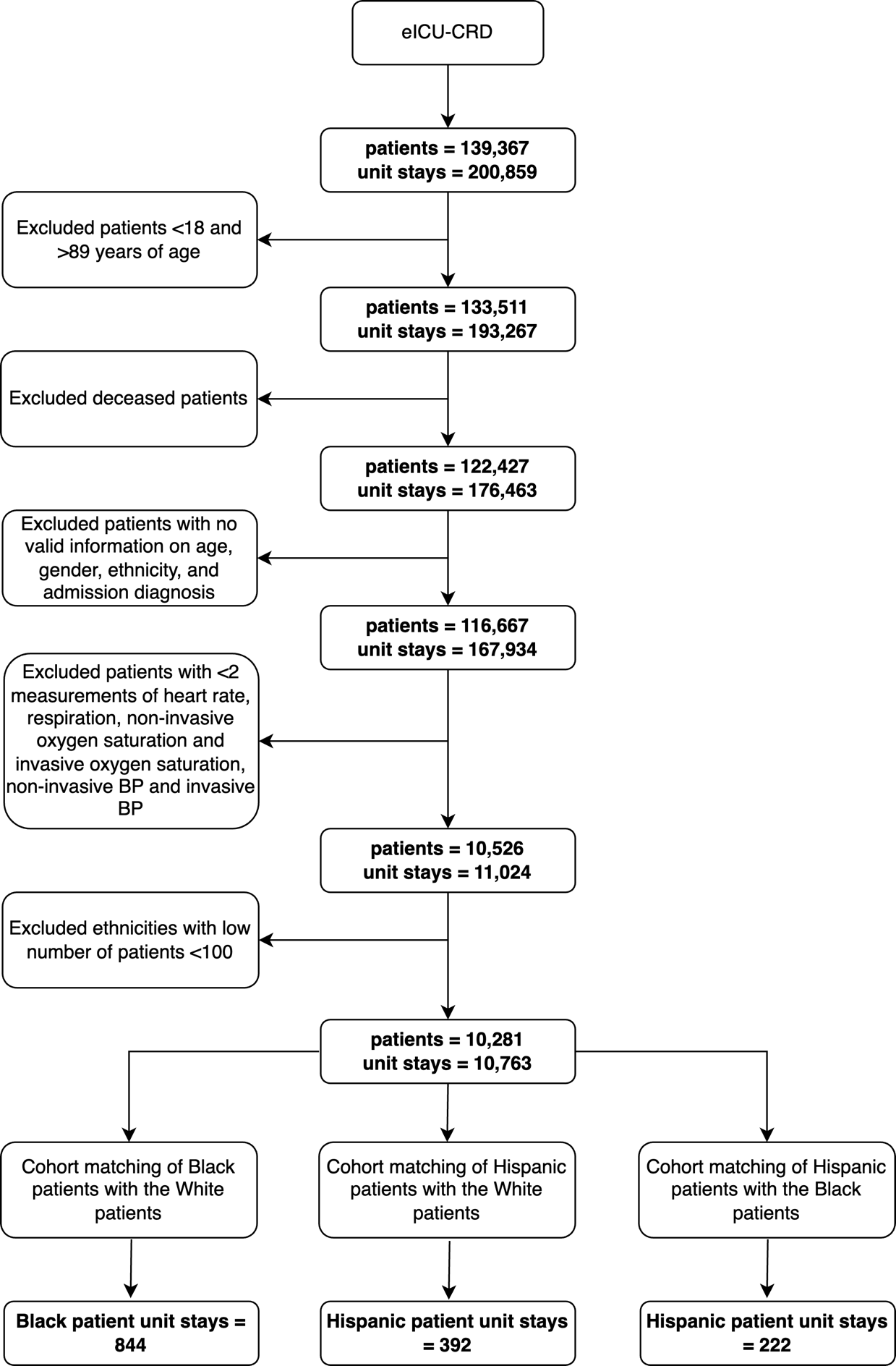


# Appendix 2 – Patient cohort matching algorithm

The original process on which the study is based matches patients with the same admission diagnosis and gender, whilst the age and APACHE IV score were matched with empirically determined ranges of ±5 and ±2.5, respectively. APACHE IV scores are included as a criterion since patients may have similar APACHE IV scores with different admission diagnoses. Cohort matching resulted in 844 Black patients matched to the same number of White patients, 392 Hispanic patients matched to as many White patients, and 222 Hispanic patients matched to as many Black patients.

To allay any concerns introduced by the matching algorithm, we have devised three additional cohorts and performed the analyses. First, 1:n matching where each patient from the lower represented group is matched to multiple patients in the higher represented group (Table A1). Second, 1:1 matching based on age, gender, and admission diagnosis, excluding APACHE IV scores (Table A2). Third, an analysis using the entire cohort derived from the selection criteria, without matching (Table A3).

*Table A1 Results obtained from 1:n matching, where patients matched within the same severity category, however without performing a 1:1 cohort matching. For the same severity category, there are many patients from the higher represented group who were matched to a single patient in the lower represented group.*

|  | Black (n=829) and White (n=2885) patients | Hispanic (n=391) and White (n=1903) patients | Hispanic (n=208) and Black (n=287) patients |
| --- | --- | --- | --- |
| Logistic Regression | 0.65 ± 0.032 | 0.71 ± 0.080 | 0.74 ± 0.042 |
| Random Forest | 0.74 ± 0.028 | 0.72 ± 0.075 | 0.73 ± 0.051 |
| XGBoost | 0.75 ± 0.029 | 0.71 ± 0.073 | 0.74 ± 0.051 |
| XGBoost (opt) | 0.76 ± 0.032 | 0.72 ± 0.067 | 0.68 ± 0.051 |

*Table A2 Results obtained from cohorts with patients matched (1:1) only on age, gender, and admission diagnosis, excluding APACHE IV scores.*

|  | Black and White patients (n=1044 per group) | Hispanic and White patients (n=475 per group) | Hispanic and Black patients (n=406 per group) |
| --- | --- | --- | --- |
| Logistic Regression | 0.73 ± 0.025 | 0.63 ± 0.042 | 0.69 ± 0.076 |
| Random Forest | 0.79 ± 0.018 | 0.71 ± 0.018 | 0.71 ± 0.062 |
| XGBoost | 0.79 ± 0.007 | 0.72 ± 0.063 | 0.72 ± 0.064 |
| XGBoost (opt) | 0.81 ± 0.011 | 0.74 ± 0.049 | 0.69 ± 0.075 |

#

*Table A3 Results obtained on the entire cohort of patients, without performing a cohort matching.*

|  | Black (n=1066) and White (n=9215) patients | Hispanic (n=482) and White (n=9215) patients | Hispanic (n=482) and Black (n=1066) patients |
| --- | --- | --- | --- |
| Logistic Regression | 0.67 ± 0.067 | 0.59 ± 0.036 | 0.73 ± 0.089 |
| Random Forest | 0.74 ± 0.044 | 0.62 ± 0.088 | 0.71 ± 0.094 |
| XGBoost | 0.73 ± 0.046 | 0.66 ± 0.110 | 0.72 ± 0.063 |
| XGBoost (opt) | 0.77 ± 0.042 | 0.67 ± 0.083 | 0.67 ± 0.084 |

# Appendix 3 – Correlation matrix between the variables used to derive the model


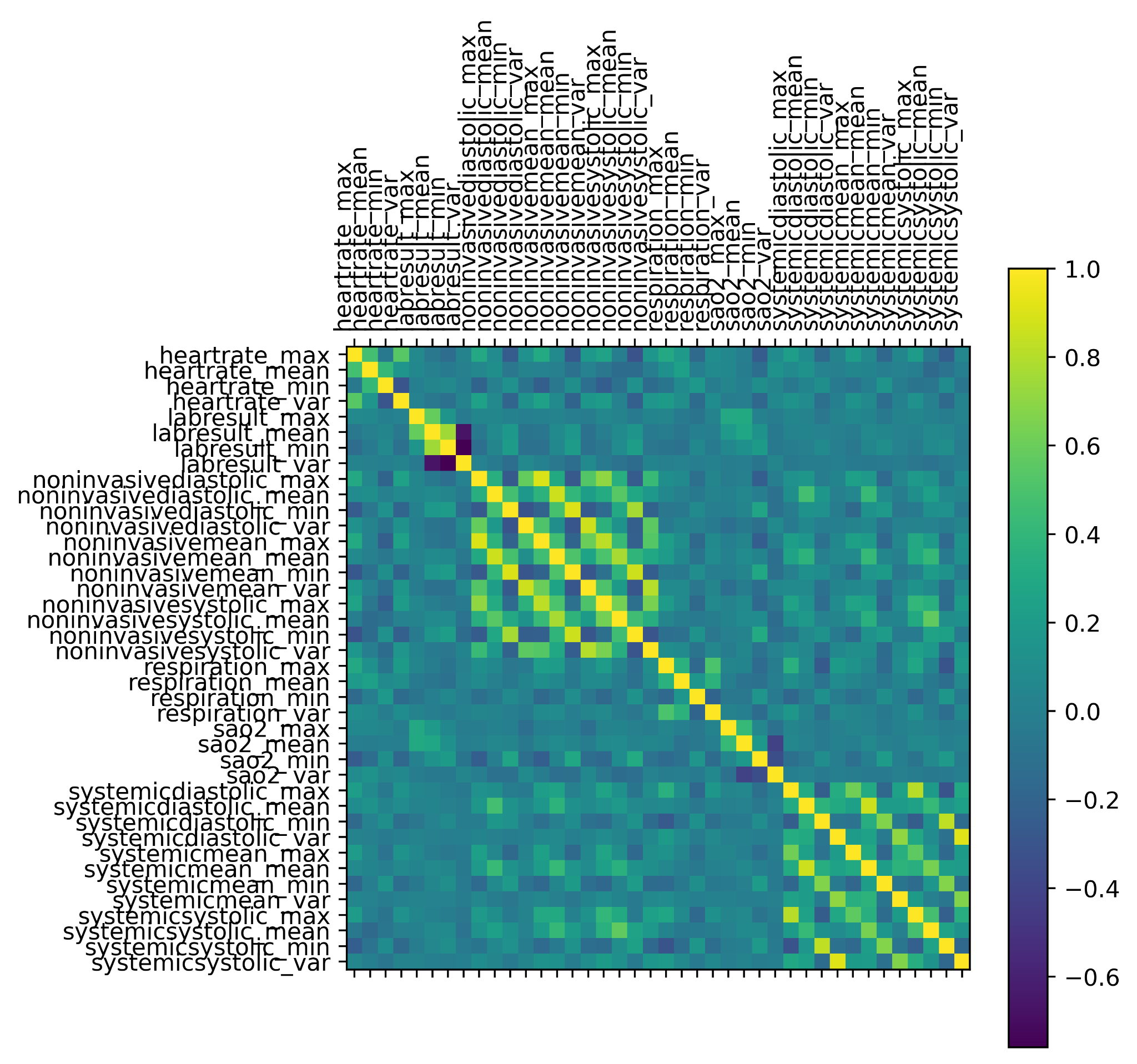


# Appendix 4 – Hyperparameter optimisation

We performed a randomised hyperparameter search optimised on a cross-validated search over the following parameter grid. The parameter optimisation was conducted in a 5-fold cross-validation setting, scoring according to the AUC metric for 500 iterations.

The analysed parameters are given in Table A4, where the parameter name is defined as per the documentation of the XGBoost classifier. The remaining three columns show the starting value of the parameter, the ending value of the parameter, and the step between considered values in the range. The obtained parameter values after the optimisation completed for each cohort are given in Table A5.

*Table A4 XGBoost parameters and their ranges used in the optimisation process*

| **Parameter** | **Start value of range** | **End value of range** | **Step value through the range** |
| --- | --- | --- | --- |
| eta | 0 | 1 | 0.05 |
| min_child_weight | 0 | 10 | 0.5 |
| gamma | 0 | 10 | 0.5 |
| subsample | 0.1 | 1 | 0.1 |
| colsample_bytree | 0 | 1 | 0.1 |
| max_depth | 2 | 7 | / |
| n_estimators | 100 | 1500 | 100 |
| learning_rate | 0.05 | 1 | 0.05 |

*Table A5 XGBoost parameters per cohort*

| **Parameter** | **Black and White patients** | **Hispanic and White patients** | **Hispanic and Black patients** |
| --- | --- | --- | --- |
| eta | 0.9 | 0.75 | 0.85 |
| min_child_weight | 5.5 | 3.5 | 1.5 |
| gamma | 9 | 9 | 8.5 |
| subsample | 0.8 | 0.5 | 0.4 |
| colsample_bytree | 0.4 | 0 | 0.5 |
| max_depth | 6 | 6 | 3 |
| n_estimators | 700 | 1400 | 700 |
| learning_rate | 0.05 | 0.05 | 0.4 |

# Appendix 5 - Patient characteristics full table

We show here the full table of patient characteristics, including the p-values which were not possible to be shown in the main manuscript due to table size requirements.

|  | **Black and White matched patient cohort** | | | **Hispanic and White matched patient cohort** | | | **Hispanic and Black matched patient cohort** | | |
| --- | --- | --- | --- | --- | --- | --- | --- | --- | --- |
| Clinical values | Black | White | p-value | Hispanic | White | p-value | Hispanic | Black | p-value |
| Patients | 844 | 844 | - | 392 | 392 | - | 222 | 222 | - |
| Gender  (male) | 457 (51.7%) | 483 (57.23%) | - | 236 (60.2%) | 238 (60.7%) | - | 141 (63.5%) | 127 (57.21%) | - |
| Age | 59 [51, 69] [19, 89] | 63 [56, 73]  [18, 89] | < 0.05 | 61 [53, 73]  [18, 89] | 64 [57, 73]  [23, 89] | < 0.05 | 62 [54, 73]  [18, 89] | 60 [52, 70]  [22, 88] | 0.151 |
| Heart rate | 87 [75, 99]  [0, 256] | 84 [73, 97]  [0, 242] | < 0.05 | 84 [73, 97]  [0, 205] | 83 [73, 96]  [0, 217] | < 0.05 | 84 [74, 96]  [0, 189] | 86 [76, 98] [0, 300] | < 0.05 |
| Invasive Oxygen Saturation | 97.6 [95, 99]  [17, 100] | 97.4 [95, 99]  [12, 100] | 0.06 | 97 [95, 99]  [43, 100] | 97 [95, 99]  [28, 100] | 0.07 | 97 [95, 99]  [54, 100] | 97 [95, 99]  [27, 100] | 0.17 |
| Oxygen Saturation (Pulse Ox) | 99 [97, 100]  [0, 100] | 98 [95, 99]  [2, 100] | < 0.05 | 98 [96, 100]  [15, 100] | 97 [95, 99]  [15, 100] | < 0.05 | 98 [96, 100]  [35, 100] | 99 [96, 100]  [0, 100] | 0.26 |
| Respiration rate | 19 [15, 24]  [0, 197] | 19 [16, 23]  [16, 189] | 0.12 | 19 [16, 23]  [0, 140] | 19 [15, 23]  [0, 147] | 0.15 | 19 [16, 23]  [0, 140] | 19 [15, 24]  [0, 152] | 0.98 |
| Invasive Systolic BP | 123 [107, 141]  [0, 300] | 120 [106, 138]  [0, 300] | < 0.05 | 122 [108, 138]  [0, 300] | 123 [108, 140]  [0, 300] | 0.25 | 119 [106, 135]  [0, 300] | 122 [108, 140]  [0, 300] | < 0.05 |
| Invasive Diastolic BP | 61 [53, 70]  [0, 300] | 58 [50, 66]  [0, 300] | < 0.05 | 59 [51, 68]  [0, 300] | 59 [51, 68]  [0, 300] | 0.28 | 58 [51, 67]  [0, 300] | 60 [53, 68]  [0, 300] | < 0.05 |
| Invasive Mean BP | 80 [72, 91]  [0, 300] | 78 [69, 87]  [0, 300] | < 0.05 | 79 [70, 90]  [0, 300] | 79 [70, 90]  [0, 300] | 0.17 | 78 [69, 88]  [0, 300] | 80 [72, 89]  [0, 300] | < 0.05 |
| Systolic blood pressure | 121 [105, 139]  [20, 287] | 118 [103, 135]  [21, 287] | < 0.05 | 120 [106, 135]  [23, 270] | 119 [105, 136]  [28, 257] | 0.31 | 117 [104, 133] [23, 270] | 123 [106, 141]  [24, 286] | < 0.05 |
| Diastolic blood pressure | 66 [57, 76]  [3, 224] | 63 [55, 73]  [0, 234] | < 0.05 | 64 [55, 74]  [10, 233] | 63 [55, 73]  [0, 219] | 0.06 | 62 [54, 72]  [10, 181] | 65 [56, 75]  [10, 210] | < 0.05 |
| Mean Arterial Pressure (MAP) | 82 [72, 94]  [9, 229] | 79 [69, 91]  [14, 269] | < 0.05 | 78 [69, 89]  [16, 238] | 80 [70, 91]  [18, 224] | < 0.05 | 76 [68, 87]  [18, 188] | 82 [72, 94]  [16, 220] | < 0.05 |
